# Does quality influence utilization of primary health care? Evidence from Haiti

**DOI:** 10.1186/s12992-018-0379-0

**Published:** 2018-06-20

**Authors:** Anna D. Gage, Hannah H. Leslie, Asaf Bitton, J. Gregory Jerome, Jean Paul Joseph, Roody Thermidor, Margaret E. Kruk

**Affiliations:** 1 Department of Global Health and Population, Harvard T.H. Chan School of Public Health, 1637 Tremont St Rm 105, Boston, MA 02120 USA; 2 Department of Health Care Policy, Harvard Medical School, Ariadne Labs, Brigham and Women’s Hospital and Harvard T.H. Chan School of Public Health, Boston, USA; 3 Zanmi LaSante, Port-au-Prince, Haiti; 4 Ministry of Health, Port-au-Prince, Haiti

**Keywords:** Health systems; primary health care; quality of care; service readiness; utilization; patient perception; Haiti

## Abstract

**Background:**

Expanding coverage of primary healthcare services such as antenatal care and vaccinations is a global health priority; however, many Haitians do not utilize these services. One reason may be that the population avoids low quality health facilities. We examined how facility infrastructure and the quality of primary health care service delivery were associated with community utilization of primary health care services in Haiti.

**Methods:**

We constructed two composite measures of quality for all Haitian facilities using the 2013 Service Provision Assessment survey. We geographically linked population clusters from the Demographic and Health Surveys to nearby facilities offering primary health care services. We assessed the cross-sectional association between quality and utilization of four primary care services: antenatal care, postnatal care, vaccinations and sick child care, as well as one more complex service: facility delivery.

**Results:**

Facilities performed poorly on both measures of quality, scoring 0.55 and 0.58 out of 1 on infrastructure and service delivery quality respectively. In rural areas, utilization of several primary cares services (antenatal care, postnatal care, and vaccination) was associated with both infrastructure and quality of service delivery, with stronger associations for service delivery. Facility delivery was associated with infrastructure quality, and there was no association for sick child care. In urban areas, care utilization was not associated with either quality measure.

**Conclusions:**

Poor quality of care may deter utilization of beneficial primary health care services in rural areas of Haiti. Improving health service quality may offer an opportunity not only to improve health outcomes for patients, but also to expand coverage of key primary health care services.

**Electronic supplementary material:**

The online version of this article (10.1186/s12992-018-0379-0) contains supplementary material, which is available to authorized users.

## Backround

Expanding coverage of maternal and child healthcare services has been a global health priority for decades and was a particular focus of the Millennium Development Goals launched in 2000 [1]. However, despite years of effort in expanding coverage, many Haitians still do not consistently utilize primary healthcare services such as antenatal and postnatal care, vaccinations, and care for sick children. In 2012, only 37% of births in Haiti were attended by skilled health personnel, 32% of newborns received postnatal care, and 38% of children with symptoms of acute respiratory tract infections were taken to an appropriate health provider [2]. Traditional healers on the other hand enjoy high use and are the first point of contact for approximately 70% of health problems [3].

Access to care in Haiti is inhibited by geographic and financial barriers, and during the time of this study (2012-2013), was also challenged by environmental and humanitarian crises such as the 2010 earthquake and subsequent cholera outbreak [4, 5]. However, there is growing evidence from around the world that poor quality of care available in health facilities may also be a significant barrier to receiving care. Poor quality can reduce utilization of healthcare services when potential users are dissuaded from seeking care due to the lack of perceived benefit or perception of harmful or unsafe care [6]. A study in Kenya identified the perceived quality of care in facilities as a strong predictor of utilization of antenatal care, immunization services, and sick child care [7]. Acharya and Cleland found that structural quality - as defined as infrastructure and availability of equipment, supplies and staff - was a greater determinant of utilization of antenatal care and Bacillus Calmette-Guerin (BCG) vaccination in Nepal than travel time to the nearest health post [8]. Beyond primary health care, discrete choice experiments in Tanzania and Ethiopia have shown that a respectful provider attitude and availability of drugs and medical equipment are important considerations to mothers deciding whether to deliver in a facility or at home [9–11].

There is evidence of poor primary healthcare quality in Haitian health facilities. Indicators of facility infrastructure are low [12], and the delivery of services is also often suboptimal. In the average primary care facility, only 8% of providers told the caregiver the diagnosis during a sick child visit and 46% of clients waited for over an hour before seeing a provider [13]. However, the association between primary healthcare quality and utilization in Haiti is unknown.

While past research establishes an important role of quality in determining utilization, it does not clearly define what aspects of quality play the largest role in utilization. Donabedian’s commonly used framework defines three broad categories for quality assessment: structure, processes, and outcomes [14]. Assessments of primary healthcare quality and utilization have largely focused on structure to the exclusion of the process and outcomes of care, defining measures of infrastructure quality composed of elements such as staffing, equipment, medicines, and amenities available at the facility [8, 12, 15]. However, patient experience and perceptions of the quality of service delivery also play a critical role in their decision to utilize care [6]. Notably, several studies on quality and utilization have used measures of patient satisfaction as proxies for quality, although these measures are subject to personal biases and may not reflect the entirety of the perceived service delivery experience [7, 16]. The quality of care experienced by an individual is likely to be multidimensional, and informed by structures, processes and outcomes. Advancing our understanding of quality and utilization therefore requires theoretically grounded measures that assess different elements of quality, enabling consideration of which elements of quality may be most important for patient decision-making.

This study addresses these gaps by combining data from facility and population-based surveys to assess the relationship between primary healthcare quality and utilization in Haiti. We compare how a measure of structural quality compares with a process-based measure of service delivery quality, and how these measures are associated with utilization of four beneficial primary healthcare services and one more complex service.

## Methods

### Study design and sample

We drew data on health service quality from the Service Provision Assessment (SPA), a census of public and private health facilities conducted in Haiti in 2013 by the Demographic and Health Surveys (DHS) Program. The SPA includes a facility assessment; interviews with healthcare providers; observations of sick child, antenatal care and family planning visits; and exit interviews with observed clients [17].

We drew data on utilization of primary care services from the 2012 DHS survey, a nationally representative household survey. As part of the two-stage sampling design, the DHS organizes communities into enumeration areas, or clusters [18]. The analytic sample includes women with a birth in the past five years and children under five in each cluster as the populations in need of maternal and child health services. Individuals who had lived in a camp since the earthquake were excluded from the analysis, as they likely sought care within the camps rather than at established health facilities. Individuals without coordinates were also excluded.

### Primary health care infrastructure and service quality

Drawing on the frameworks of care quality described above, we examined two quality dimensions. First, infrastructure of healthcare facilities was measured using the service readiness index (SRI) from the WHO general service readiness measure [19]. It comprises 48 indicators in five domains: essential medicines; diagnostic capacity; basic amenities; infection prevention; and basic equipment. The full list of indicators is in Additional file 1. The domain scores are calculated and then averaged across each facility to obtain a single service readiness score between 0 and 1.

Second, we quantified service delivery quality using a measure adapted from the Primary Health Care Performance Initiative (PHCPI) framework as described in Gage et al. [13, 20]. The framework delineates four domains of primary health care service delivery performance: accessible care (i.e. timeliness); effective service delivery (i.e. provider competence); management and organization (i.e. information system use); and primary health care functions (i.e. person-centered care). The measure encompasses 28 indicators, listed in Additional file 1, from the SPA facility assessment, provider interview, observations of care and exit interviews that match each quality sub-domain. These domains include objectively measured indicators (i.e. the clients saw providers for at least 15 minutes each), and patient’s experience of quality (i.e. how the staff treated the client was a problem). We created a score for each domain from the mean of all the indicators in that domain, and we averaged the four scores together to create a single overall measure of the facility’s service delivery quality for primary care between 0 and 1.

Although the SPA was a census of facilities, clinical observations and patient exit interviews were conducted only in a subset of facilities, which affected 12 of the 28 indicators in the service delivery quality score. To address missing data, we used covariates such as facility and management type and urban to impute the missing values of the indicators in five completed datasets. In each dataset, we calculated the service delivery index with the observed and imputed indicators and used multiple imputation estimates to assess measure variance.

### Primary health care utilization

We examined the association of quality with utilization of four primary care services, focusing on the key areas of maternal and child care that are available in the DHS survey: antenatal care (ANC), postnatal care (PNC), vaccination and sick child visits. We included two indicators of ANC utilization: any ANC visit and the minimum recommended four visits (complete ANC) with a qualified provider during the most recent pregnancy for women with a birth in the last five years. PNC utilization was defined as mothers reporting a newborn check received within six weeks of birth among women with a birth in the last five years. To account for the time lag between utilization and quality measurement, we assessed antenatal and postnatal utilization for pregnancies in the last two years in a sensitivity analysis. Complete vaccination was calculated as receipt of BCG vaccine plus three diphtheria, pertussis and tetanus (DPT) vaccines and three polio and measles vaccines among children between one and five years old. Sick child utilization was defined as children who went to a facility for treatment among all children under age five with diarrhea, fever or cough in the two weeks preceding the survey. In addition to the primary care services, we also examined facility delivery rate as a falsification test with our measure of primary care service delivery.

### Covariates

Drawing on Andersen’s behavioral model of health care utilization, we selected covariates that might influence quality of care as well as utilization [21]. Included covariates are whether the cluster is urban or rural, household poverty (belonging in the lowest two wealth quintiles), woman’s education level, whether the woman is married or cohabitating, and woman’s age.

### Linking clusters to facilities

There are several challenges in linking population surveys with health facility surveys [22]. First, the DHS survey displaces the geographic coordinates of the population data in order to protect the privacy of the respondents. Second, the DHS survey does not collect detailed information on what health facility the respondent would regularly use in a way that corresponds with facility data gathered in SPA. To address these challenges, we considered two methods for linking the population to facilities. Our primary method was to define a service environment around each cluster [12]. We defined service environments for each service of interest: ANC, vaccination, sick child care and delivery. If a facility provided ANC, we assume they also provided PNC [23]. In rural clusters, the service environment was defined as all facilities that provided the service of interest with 5 km of a cluster; in urban clusters it was 2 km, according to DHS guidelines [24]. Buffers of 10 km and 5 km for rural and urban clusters respectively as a sensitivity check. If there was no facility providing the service within the buffer, the nearest facility offering that service within 10 km was the cluster’s service environment. Clusters that were not within 10 km of the service were excluded from the analysis [4]. The facilities composing the service environment may therefore vary by service. All facilities providing primary health care services were included in the analysis, regardless of whether they were considered primary care facilities, as primary care is still provided in hospital settings. The quality scores of all facilities in the service environment were averaged to describe the level of quality available in the surrounding area. For facilities that required imputation of individual quality items, we used the average of the 5 imputation-based quality scores in calculating the service environment average.

We also considered the hypothesis that utilization is not driven by the average quality of all nearby facilities, but rather by the quality of the best performing facility in the area: if one facility was known for exceptional quality it may drive demand in its area. In our secondary linking method, we therefore selected the highest quality score from the same service environment and assigned that quality to the cluster.

All distances used for linking clusters to facilities were linear and did not account for topography or road network, a reasonable approach in past studies [25].

### Analysis

Because there are several differences between rural and urban areas in distance, number of surrounding facilities, and health seeking behavior, the analysis is stratified by location. We calculated descriptive statistics of the population in need of primary health care services and facility characteristics. We examined the association between each quality dimension and the probability of seeking care using a multilevel log binomial model with individuals clustered within their communities and robust standard errors. The ANC utilization and urban facility delivery models did not converge with the log binomial; a Poisson model was instead used to estimate the relative risk [26]. To illustrate selected results, we used the regression models to predict utilization across the range of quality scores in rural clusters, holding covariates at their means. The descriptive statistics use sampling weights, while the regressions are unweighted.

Multiple imputation was conducted in R 3.3; all other analyses were conducted in Stata 14.0.

## Results

The DHS gathered data on 4,974 women with a pregnancy in the last five years and 6,263 children younger than 5 years old in Haiti. Individual characteristics and service utilization are displayed in Table 1. Approximately two thirds of the sample lived in rural areas. Wealth and education were concentrated in urban areas; less than 1% of urban women were in the bottom two wealth quintiles nationally. Care utilization was also higher in urban areas for nearly all examined services. Access to care was highest for any ANC: 90% of urban pregnant women and 86% of rural pregnant women received at least one visit. Sick child care was the least utilized service in urban areas, while facility delivery was the least utilized in rural areas.

**Table 1:** Characteristics of women and children in clusters

	Urban		Rural	
	(*N* women = 1668)		(*N* women = 3230)	
	*N*	%	*N*	%
Service utilization for most recent pregnancy				
Any ANC care	1432	90%	2543	86%
Complete ANC care	1195	75%	1757	59%
Facility delivery	972	60%	772	26%
Any postnatal care	713	45%	1304	44%
Service utilization for children under 5				
Complete vaccinations for age	729	46%	1451	44%
Sick child visit	459	41%	692	31%
Community characteristics				
Women’s education level				
No education	398	7%	1669	22%
Primary	1506	28%	3268	44%
Secondary	2923	55%	2393	32%
Higher	526	10%	144	2%
Lowest two wealth quintiles	51	1%	4218	60%
Married or cohabitating	2670	50%	4080	58%
Women’s age (Mean, SD)	27.9	9.3	28.4	10.0

The SPA obtained detailed data on 905 out of the country’s 907 facilities. Table 2 displays the service availability and quality scores of the facilities. Nearly all facilities provided ANC and sick child services: 95% and 97% of rural facilities respectively. Normal delivery services were available in only 44% of facilities in rural and urban areas. Urban facilities had much higher infrastructure scores than in rural areas, scoring 0.62 versus 0.50 out of one. Basic equipment such as scales and a stethoscope were generally available at facilities, scoring 0.82 out of 1. Facilities scored lowest on diagnostic capacity (0.34), lacking items such as a urine pregnancy test. The average facility service delivery score was 0.58, though the variation was much smaller than for infrastructure: service delivery scores ranged from 0.47 to 0.81 compared to a range of 0.21 to 0.96 for infrastructure across service environments. The distribution of infrastructure and service quality for the sick child service environment, the most widely available service, is depicted in Additional file 1. The two measures of quality were positively correlated in facilities (Pearson’s correlation coefficient = 0.32).

**Table 2:** Facility characteristics (N=907)

	Urban (*N*= 354)		Rural (*N*=553)	
	N/Mean	%/SD	N/Mean	%/SD
Service availability				
Antenatal care	310	88%	522	95%
Delivery	155	44%	240	44%
Sick child	315	89%	533	97%
Vaccinations	245	69%	436	79%
Facility quality				
Infrastructure (SRI)	0.62	0.15	0.50	0.15
Service delivery (PHPCI)	0.57	0.09	0.58	0.08

Tables 3 and 4 show the association between utilization and the average service environment quality for each service from the multilevel analysis. There was a positive association between facility quality and utilization for more services in rural areas than urban. In urban areas, infrastructure quality was positively associated with PNC utilization; no other association was significant. In rural areas, infrastructure quality was positively associated with ANC, facility delivery, PNC and vaccinations. The association was strongest for PNC utilization and vaccination utilization (RR= 2.31, CI: 1.43, 3.74 and RR = 1.81, CI: 1.23, 2.67). Service delivery scores were significantly associated with ANC, PNC and vaccinations in rural areas. Facility delivery was not associated with primary care service delivery quality. The magnitudes of these associations were much larger than those with infrastructure scores; the adjusted relative risk of vaccination utilization from improved service delivery is 5.44 (CI: 1.84,16.00). Sick child utilization was not significantly associated with either measure of quality. Additional file 1 displays the full output from all models.

**Table 3:** Association between service utilization and quality in urban areas, adjusting for poverty, education, married, age, age squared

	Infrastructure (SRI)			Service delivery (PHCPI)		
	RR^a^	p	*N*	RR^a^	p	*N*
Any ANC	0.95	0.56	1618	1.14	0.34	1618
Complete ANC	0.82	0.24	1618	0.98	0.94	1618
PNC	*3.06*	*0.00*	1618	0.72	0.64	1618
Complete vaccinations	1.84	0.07	1636	2.56	0.07	1636
Sick child visit	1.00	1.00	1126	1.12	0.85	1126
Facility delivery	1.48	0.16	1461	0.87	0.80	1461

^a^Risk ratio for the log binomial models and rate ratio for Poisson models (ANC and facility delivery)

Italicized values have *p*<0.05

**Table 4:** Association between service utilization and quality in rural areas, adjusting for poverty, education, married, age, age squared

	Infrastructure (SRI)			Service delivery (PHCPI)		
	RR^a^	p	*N*	RR^a^	p	*N*
Any ANC	*1.30*	*0.00*	3230	*1.99*	*0.00*	3230
Complete ANC	*1.44*	*0.02*	3230	*2.33*	*0.01*	3230
PNC	*2.31*	*0.00*	3230	*3.11*	*0.03*	3230
Complete vaccinations	*1.81*	*0.00*	3601	*5.44*	*0.00*	3601
Sick child visit	0.94	0.80	2545	1.78	0.42	2545
Facility delivery	*1.78*	*0.02*	3236	0.73	0.63	3236

^a^Risk ratio for the log binomial models and rate ratio for Poisson models (ANC)

Italicized values have *p*<0.05

**Fig. 1:**
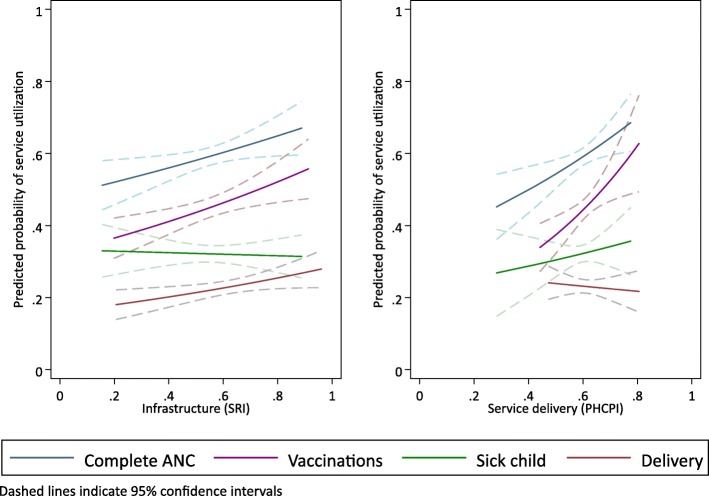
Predicted service utilization by infrastructure quality (SRI) and service delivery (PHCPI) scores in rural areas

Figure 1 displays the predicted utilization of complete ANC, facility delivery, sick child services, and complete vaccinations in rural areas from the range of infrastructure and service delivery scores based on the regressions from Tables 3 and 4. Quality was more strongly associated with higher utilization for services with higher baseline utilization. The magnitude of association is larger for service delivery quality for complete ANC and vaccination utilization than the corresponding associations with infrastructure scores. For completed vaccination rate, a change in average infrastructure scores in the service environment from 0.50 to 0.75 corresponds to a predicted change from 49% to 57% complete vaccination, while the same change in service environment service delivery would result in a predicted change from 42% to 61%.

The results of the regressions using the alternate linking mechanism are shown in Additional file 1. Though it varied by service, highest performing facilities in a cluster were typically private primary care facilities. The association between utilization and quality was weaker when using the highest performing facility in the service environment rather than the average service environment quality, though ANC and PNC utilization were still significantly associated. Sensitivity analyses using the larger buffer distances and antenatal and postnatal utilization in the past two years are also available in Additional file 1. The findings were robust to the larger buffer sizes; however, the sample sizes were small when limited to women with pregnancies in the past two years, therefore fewer associations were significant in the second analysis.

## Discussion

This analysis examines how health service quality may influence community members’ decisions to seek care at a given facility. Aggregate quality of primary health care provided in Haitian facilities in 2013 was low; on average, facilities met just over half of the basic standards for infrastructure and for service delivery quality. In rural areas, the quality of primary health care service delivery was more strongly associated with utilization of several key services than the quality of facility inputs and infrastructure. Service delivery may have had a larger impact than inputs because it is a more comprehensive measure of the care experience, including indicators such as wait time, availability of services, provider competence, duration of visit and adequate communication with the provider. These results suggest patients perceive structural and process measures of quality, and consider them both when deciding whether and where to utilize care. Though the results were mixed, services with higher baseline utilization appeared to be more strongly associated with both types of quality, suggesting that as exposure to the service increases, clients may be able to better discern the quality of care.

Both infrastructure and service delivery quality in our study were more strongly associated with primary health care utilization in rural areas than in urban areas. The weak association between quality and utilization in urban areas may be due to several factors. First, urban dwellers had many more facility options in a geographically concentrated area and greater transportation options, so may not be accessing the actual facilities in the service areas we designated [27]. To compensate for the additional distance and other barriers that rural dwellers face, they may require greater value from the service in order to seek care. However, urban dwellers’ utilization decisions may also have been motivated by higher expectations of quality than rural dwellers that are not captured by these basic quality standards [28]. Sparse observations at the higher end of the quality spectrum limit our ability to understand whether there may be a quality threshold above which urban dwellers would be willing to seek care. More research is therefore required to determine whether the lack of association is valid or due to linking challenges. PNC was associated with infrastructure quality in urban areas, though more context-specific research is also required to verify this association and understand how PNC may differ from other services. The association between quality and utilization was weaker when we linked the cluster to the best performing facility in the region rather than the average. This may be because the communities may not be able to shop around for the best facility in their area or the top facility’s capacity is limited.

Sick-child care utilization was not associated with either infrastructure or service delivery quality in urban or rural areas. Sick-child care differs from the other primary health care services considered in that it is a curative service for an acute need rather than a preventive, routine service. As such, it requires more complex diagnostic and therapeutic modalities that integrate availability of inputs, availability of competent providers, and motivation to carry out treatment plans. Whereas the immediate service environment may suffice for routine services, a parent may consider a more distant facility with perceived higher quality, such as a hospital, to get care when a child becomes ill. Therefore, the quality of the nearest facility may not determine utilization decisions. Alternately, parents might seek the nearest facility irrespective of quality, especially when the child is very ill. Several studies in lower- and middle-income countries have found that caregiver’s decision to seek care for their child may be more determined by the severity of illness irrespective of the quality of care at any facility [29, 30].

Though there are several indicators on the patient’s perceptions in the service delivery quality index, all infrastructure quality and most service delivery quality indicators are objectively measured. We therefore assume that utilization is associated with quality in rural areas because the users are able to discern higher quality facilities and vote with their feet to get to the better value. Other studies confirm that users are able accurately judge the quality of care provided [31–33]. This judgement matters: evidence from high income countries found that a patient’s own previous experience is for many patients the most important information source on care [34]. This suggests that intervening at the health system to improve patient’s perceptions of care may be more effective than external campaigning to encourage people to seek care.

Our findings align with those of Wang et al, who found that infrastructure quality was associated with utilization of several primary care services, particularly in rural areas of Haiti [12]. However, Wang et al also found an association between facility childbirth delivery and infrastructure quality that was not found in this study. Our analysis diverges from this study in a number of ways. In particular, the earlier paper used a measure of infrastructure quality more specific to obstetric services, rather than our more comprehensive facility readiness measure. As a more complex service, facility delivery is not considered a part of primary care, and therefore we hypothesized that it would not be associated with primary care quality [35]. Our results supported this hypothesis: while facility delivery was associated with infrastructure quality in rural areas, it was not significantly associated with the service delivery quality, lending credence that the service delivery measure is capturing primary care quality.

This is one of few studies to link a national population survey to a health facility census to understand in more detail how people interact with the local health system. We move beyond a narrow structural view of health care quality to consider how well care was provided, and begin to assess how the perceived experience of that care may influence utilization decisions. In looking at measures of both infrastructure quality and service delivery quality, we find that service delivery quality is more strongly associated with utilization than simple infrastructure availability. While we conceptualized process quality as a single multidimensional measure, future research may also investigate what service delivery quality domains (i.e. effective service delivery or management and organization) have the greatest associations with utilization.

This study has several limitations. Due to geographic displacement and lack of road network or topography data, facilities in the service environment may not be the population’s true set of facility options. Further, the surveys were done roughly one year apart, so people and providers may have moved, particularly in the continued aftermath from the disruptive earthquake and cholera outbreak. A mismatch of communities and service environments would likely result in an underestimate of the true association between utilization and quality, as is particularly likely in urban areas. Population surveys must collect better concurrent data on which facilities (e.g. type and relative proximity) the households consider their usual source of care in order to more appropriately and temporally link population and health facility datasets. These data would enable analyses measuring the association between facility quality and many other outcomes of quality, including health outcomes. Although we used multiple imputation to fill in missing data for the service delivery scores where observations of clinical visits were not conducted, we were unable to adequately account for this uncertainty in defining the service environment scores. However, because data were available for the majority of service delivery indicators prior to averaging within facility and within service environment, we expect this would have a negligible impact on the associations observed. In addition, as a cross-sectional study, we cannot infer causation between quality and utilization.

There are several implications of this work for Haiti’s primary health care system. First, the low correlation between the measures of infrastructure and service delivery quality at facilities suggest that poor service delivery can be provided in the presence of high quality inputs and vice versa. Better infrastructure does not necessarily equate to better technical or interpersonal quality;[36] rather, both inputs and processes must be improved and integrated at the point of service delivery to realize the desired outcomes, especially for more complex diagnostic and therapeutic tasks. Second, the study suggests that better service quality may encourage people to utilize more primary health care particularly in rural areas, thus expanding effective coverage of important services. While utilization was higher for some services near high quality facilities, both infrastructure and service delivery quality was on average still very poor throughout the country. Improving the currently inadequate levels of primary health care quality in facilities should therefore be a priority for the Ministry of Health. Doing so will require a focus on strengthening the process of care underlying service delivery.

Improving access to, and quality of, primary health care need not be mutually exclusive goals. Indeed, our study suggests they are mutually reinforcing. Quality efforts can build upon the government and non-government initiatives that have focused on improving access to primary care in Haiti in the past 20 years, such as a performance-based financing initiative which resulted in significant increases in infant, under-five and antenatal care visits [37]. Other programs have lowered barriers to using antenatal care and facility delivery and integrated HIV and TB prevention and treatment into primary care to scale up access to antenatal care and vaccine administration [38–40]. These programs complement those targeting quality of care including the National Quality Committee and regular quality measurement in the national electronic medical record system [41].

## Conclusions

Health system quality may be an underappreciated lever for influencing utilization of primary care services. The associations found in rural Haiti in this analysis suggest that quality improvements could act synergistically to increase utilization and provide more effective care. A more granular understanding of health system utilization and quality of care is necessary to build on this research, particularly in urban areas.

## Additional file

Additional file 1: Supplemental results. (DOCX 10906 kb)

## Abbreviations

ANC: Antenatal care
BCG: Bacillus Calmette-Guerin
CI: Confidence interval
DHS: Demographic and Health Surveys
DPT: Diphtheria, Pertussis and Tetanus
PHCPI: Primary Health Care Performance Initiative
PNC: Postnatal care
RR: Relative risk
SPA: Service Provision Assessment
SRI: Service Readiness Index
WHO: World Health Organization
