# Supplementary material for: Does quality influence utilization of primary health care? Evidence from Haiti
Source: Global Health. 2018 Jun 20;14:59. doi: 10.1186/s12992-018-0379-0 (PMC6011404; doi:10.1186/s12992-018-0379-0)
Supplement: Supplementary file 1 — Supplemental results. (DOCX 10906 kb) [file 12992_2018_379_MOESM1_ESM.docx]

Appendix 1: Quality measure components

Service readiness index measure components

| Domain | Indicator | Mean | SD |
| --- | --- | --- | --- |
| Basic amenities | Central electricity or functional generator with fuel | 0.52 | 0.50 |
|  | Improved water source available year-round within 500 meters | 0.42 | 0.49 |
|  | Auditory and visual privacy in exam area | 0.96 | 0.20 |
|  | At least one functional client toilet observed | 0.46 | 0.50 |
|  | Facility phone or short-wave radio available at all times | 0.67 | 0.47 |
|  | Computer with internet | 0.30 | 0.46 |
|  | Functional ambulance with fuel | 0.12 | 0.32 |
| Basic equipment | Functional adult scale | 0.92 | 0.28 |
|  | Functional pediatric scale | 0.65 | 0.48 |
|  | Functional thermometer | 0.96 | 0.19 |
|  | Functional stethoscope | 0.99 | 0.12 |
|  | Functional blood pressure apparatus | 0.98 | 0.14 |
|  | Functional light | 0.44 | 0.50 |
| Infection prevention | Sharps are adequately disposed of | 0.63 | 0.48 |
|  | Medical or contaminated waste is adequately disposed of | 0.56 | 0.50 |
|  | Sharps box available | 0.92 | 0.26 |
|  | Waste bin available | 0.35 | 0.48 |
|  | Surface disinfectant available | 0.86 | 0.35 |
|  | New syringes available | 0.94 | 0.23 |
|  | Water and soap or hand disinfectant available | 0.86 | 0.35 |
|  | Gloves available | 0.95 | 0.21 |
|  | At least one guideline for infection control observed | 0.37 | 0.48 |
| Diagnostics | Functional haemoglobin / anemia testing | 0.07 | 0.26 |
|  | Functional blood glucose testing | 0.48 | 0.50 |
|  | Functional malaria testing | 0.68 | 0.47 |
|  | Functional urine dipstick test for protein | 0.48 | 0.50 |
|  | Functional urine dipstick test for glucose | 0.46 | 0.50 |
|  | Functional HIV testing | 0.47 | 0.50 |
|  | Functional syphilis testing | 0.47 | 0.50 |
|  | Functional urine dipstick test for pregnancy | 0.61 | 0.49 |
| Medicines: observed and valid | Amitriptyline tablets | 0.04 | 0.19 |
|  | Amlodipine tablet or alternative Ca channel blocker | 0.19 | 0.39 |
|  | Amoxicillin syrup | 0.76 | 0.43 |
|  | Amoxicillin [ampicillin] | 0.78 | 0.42 |
|  | Ampicillin powder for injection | 0.29 | 0.45 |
|  | Beclomeasone inhaler | 0.04 | 0.19 |
|  | Ceftriaxone injection | 0.30 | 0.46 |
|  | Enalapril tablet or alternative ACE inhibitor | 0.56 | 0.50 |
|  | Gentamicin injection | 0.29 | 0.45 |
|  | Glibenclamide tablets | 0.27 | 0.45 |
|  | Insulin injection | 0.10 | 0.30 |
|  | Metformin tablets | 0.41 | 0.49 |
|  | Omeprazole or alternative *prazole tab | 0.50 | 0.50 |
|  | Oral rehydration salts | 0.24 | 0.43 |
|  | Paracetamol tablets | 0.76 | 0.43 |
|  | Salbutamol inhaler | 0.36 | 0.48 |
|  | Simvastatin or other statin | 0.09 | 0.29 |
|  | Zinc tablet or syrup | 0.54 | 0.50 |

Primary health care performance index measures components

| Domain | Indicator | Source | Mean | SD |
| --- | --- | --- | --- | --- |
| Accessible care | Financial: Cost was not a problem for clients | Exit interview | 0.93 | 0.16 |
|  | Financial: Proportion of five primary care services without fees | Facility assessment | 0.48 | 0.31 |
|  | Timeliness: Clients wait less than an hour | Exit interview | 0.44 | 0.39 |
|  | Timeliness: Wait time was not a problem for clients | Exit interview | 0.81 | 0.24 |
| Effective service delivery | Provider availability: Percentage of providers working full time | Provider interview | 0.58 | 0.40 |
|  | Provider availability: Percentage of days/month on which primary health services offered | Facility assessment | 0.62 | 0.20 |
|  | Provider competence: Asked maternal age during first ANC visit | Clinical observations | 0.54 | 0.40 |
|  | Provider competence: Took child's temperature during sick child visit | Clinical observations | 0.82 | 0.30 |
|  | Provider motivation: Providers have opportunity for promotion | Provider interview | 0.20 | 0.27 |
|  | Provider motivation: Providers have written job descriptions | Provider interview | 0.30 | 0.34 |
|  | Provider motivation: Clients saw providers for at least 15 minutes each | Clinical observations | 0.37 | 0.34 |
|  | Patient-provider respect: Treatment by staff not a problem for clients | Exit interview | 0.97 | 0.12 |
|  | Patient-provider respect: Consultation areas have auditory and visual privacy | Facility assessment | 0.75 | 0.43 |
|  | Safety: Appropriate waste disposal | Facility assessment | 0.50 | 0.28 |
|  | Safety: Proportion of rooms with infection control items | Facility assessment | 0.36 | 0.45 |
| Management and organization | Facility management capacity: Appropriate management meetings | Facility assessment | 0.30 | 0.29 |
|  | Supportive supervision: External supervision in previous six months | Facility assessment | 0.87 | 0.34 |
|  | Supportive supervision: Proportion of providers with recent supervision | Provider interview | 0.72 | 0.34 |
|  | Information system use: HMIS and trained personnel | Facility assessment | 0.90 | 0.30 |
|  | Quality improvement: Facility gathers client feedback | Facility assessment | 0.03 | 0.17 |
|  | Quality improvement: Routine quality assurance | Facility assessment | 0.31 | 0.46 |
| Primary care functions | First-contact accessibility: Sick child did not visit traditional healer first | Exit interview | 0.97 | 0.10 |
|  | Continuity: Caregivers would return to facility with child | Exit interview | 0.70 | 0.29 |
|  | Continuity: Proportion of services with client records maintained | Facility assessment | 0.61 | 0.33 |
|  | Comprehensiveness: care available for NCDs | Facility assessment | 0.62 | 0.39 |
|  | Comprehensiveness: Maternal and child health services available | Facility assessment | 0.76 | 0.24 |
|  | Comprehensiveness: Infectious disease services available | Facility assessment | 0.72 | 0.26 |
|  | Coordination: Communication capability and ambulance | Facility assessment | 0.50 | 0.37 |
|  | Coordination: Proportion of services with test results register | Facility assessment | 0.46 | 0.57 |
|  | Person-centered care: ANC clients told adverse effects of iron supplements | Clinical observations | 0.03 | 0.14 |
|  | Person-centered care: Caregivers told their sick children's diagnoses | Clinical observations | 0.09 | 0.21 |
|  | Person-centered care: Patients very satisfied with care | Exit interview | 0.93 | 0.15 |

Appendix 2. Sick child service environment quality scores by cluster


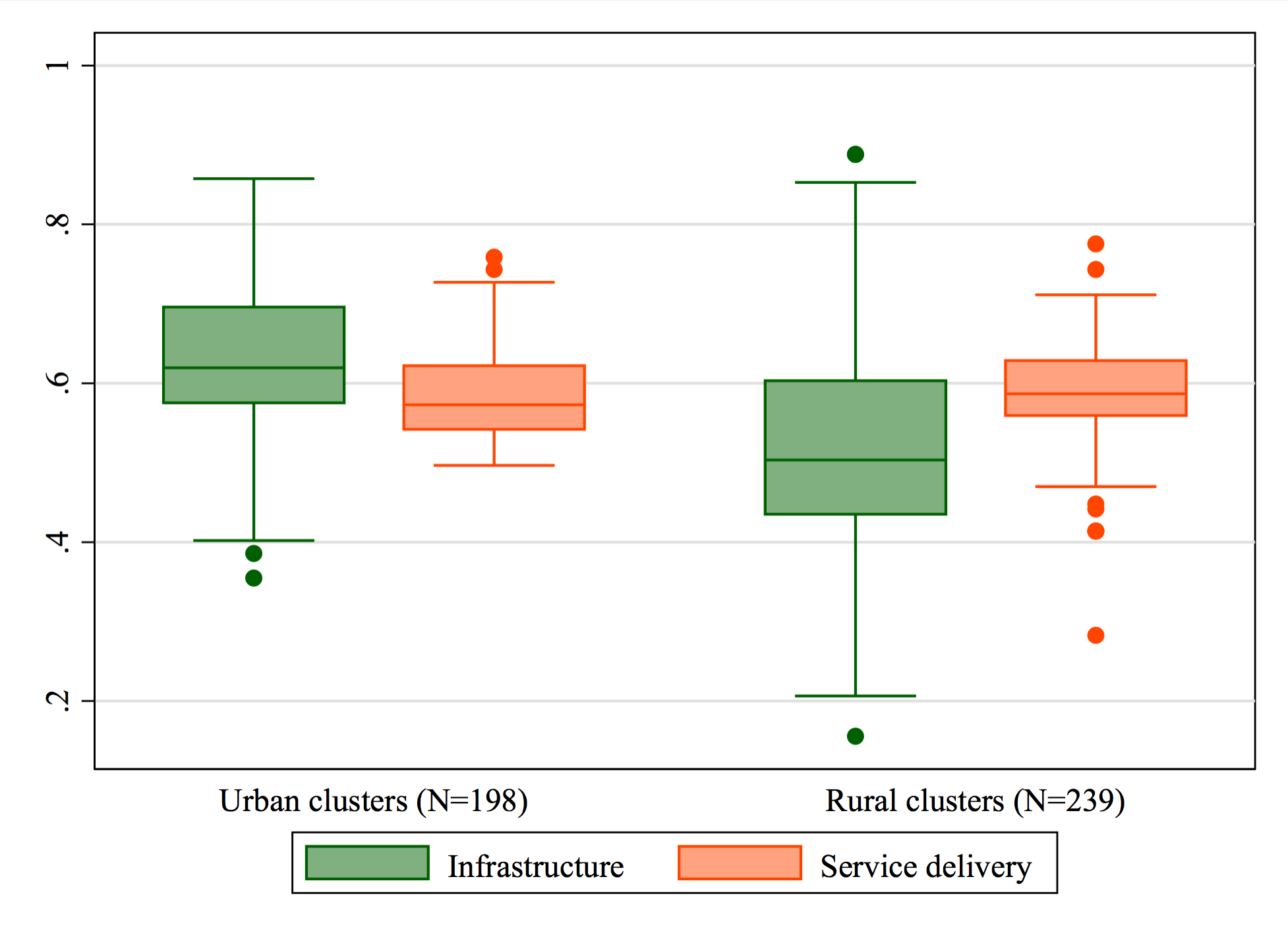


Appendix 3. Full model output from Tables 3 and 4

| SRI quality and utilization in urban households | | | | |  |  |  |  |  |  |  |  |
| --- | --- | --- | --- | --- | --- | --- | --- | --- | --- | --- | --- | --- |
|  | Any antenatal care | | Complete antenatal care | | Postnatal care | | Facility-based delivery | | Vaccinations | | Sick child visit | |
|  | RR | p | RR | p | RR | p | RR | p | RR | p | RR | p |
| SRI quality | 0.95 | 0.56 | 0.82 | 0.24 | 3.06 | 0.002** | 1.48 | 0.16 | 1.84 | 0.07 | 1.00 | 1.00 |
| Poverty | 0.87 | 0.08 | 0.83 | 0.12 | 0.96 | 0.79 | 0.72 | 0.06 | 0.68 | 0.09 | 1.00 | 0.99 |
| Married or cohabitating | 1.02 | 0.45 | 1.04 | 0.30 | 1.09 | 0.21 | 0.94 | 0.25 | 1.11 | 0.22 | 0.93 | 0.48 |
| Age | 0.99 | 0.28 | 1.03 | 0.14 | 0.99 | 0.70 | 0.97 | 0.31 | 1.12 | 0.001** | 1.07 | 0.21 |
| Age Squared | 1.00 | 0.26 | 1.00 | 0.28 | 1.00 | 0.57 | 1.00 | 0.25 | 1.00 | 0.006** | 1.00 | 0.22 |
| Women's education |  |  |  |  |  |  |  |  |  |  |  |  |
| No education | Ref |  | Ref |  | Ref |  | Ref |  | Ref |  | Ref |  |
| Primary | 1.17 | 0.004** | 1.31 | 0.000*** | 1.08 | 0.47 | 1.13 | 0.31 | 1.28 | 0.06 | 1.48 | 0.031* |
| Secondary | 1.27 | 0.000*** | 1.62 | 0.000*** | 1.11 | 0.33 | 1.77 | 0.000*** | 1.47 | 0.003** | 1.66 | 0.004** |
| Higher | 1.31 | 0.000*** | 1.80 | 0.000*** | 1.29 | 0.049* | 2.45 | 0.000*** | 1.66 | 0.000*** | 2.03 | 0.001** |
| Constant | 0.88 | 0.42 | 0.34 | 0.000*** | 0.21 | 0.001** | 0.44 | 0.036* | 0.68 | 0.09 | 1.00 | 0.99 |
| N | 1618 |  | 1618 |  | 1618 |  | 1461 |  | 1636 |  | 1126 |  |
|  |  |  |  |  |  |  |  |  |  |  |  |  |
| SRI quality and utilization in rural households | | | | |  |  |  |  |  |  |  |  |
|  | Any antenatal care | | Complete antenatal care | | Postnatal care | | Facility-based delivery | | Vaccinations | | Sick child visit | |
|  | RR | p | RR | p | RR | p | RR | p | RR | p | RR | p |
| SRI quality | 1.30 | 0.004** | 1.44 | 0.021* | 2.31 | 0.001*** | 1.78 | 0.024* | 1.81 | 0.003** | 0.94 | 0.80 |
| Poverty | 0.97 | 0.044* | 0.82 | 0.000*** | 0.97 | 0.56 | 0.56 | 0.000*** | 0.92 | 0.12 | 0.79 | 0.002** |
| Married or cohabitating | 1.01 | 0.49 | 1.05 | 0.27 | 1.11 | 0.08 | 1.02 | 0.81 | 0.96 | 0.46 | 1.08 | 0.41 |
| Age | 1.02 | 0.042* | 1.05 | 0.002** | 1.06 | 0.003** | 0.95 | 0.06 | 1.07 | 0.003** | 0.98 | 0.46 |
| Age Squared | 1.00 | 0.040* | 1.00 | 0.006** | 0.00 | 0.004** | 1.00 | 0.11 | 1.00 | 0.008** | 1.00 | 0.61 |
| Women's education |  |  |  |  |  |  |  |  |  |  |  |  |
| No education | Ref |  | Ref |  | Ref |  | Ref |  | Ref | Ref | Ref | Ref |
| Primary | 1.11 | 0.000*** | 1.23 | 0.000*** | 1.12 | 0.036* | 1.69 | 0.000*** | 1.16 | 0.005** | 1.19 | 0.06 |
| Secondary | 1.17 | 0.000*** | 1.48 | 0.000*** | 1.30 | 0.000*** | 2.97 | 0.000*** | 1.27 | 0.000*** | 1.53 | 0.000*** |
| Higher | 1.17 | 0.001*** | 1.61 | 0.000*** | 1.57 | 0.005** | 4.50 | 0.000*** | 1.51 | 0.014* | 1.56 | 0.29 |
| Constant | 0.55 | 0.000*** | 0.19 | 0.000*** | 0.09 | 0.000*** | 0.31 | 0.015* | 0.10 | 0.000*** | 0.47 | 0.13 |
| N | 3230 |  | 3230 |  | 3230 |  | 3236 |  | 3601 |  | 2545 |  |
|  |  |  |  |  |  |  |  |  |  |  |  |  |
| PHCPI quality and utilization in rural households | | | | |  |  |  |  |  |  |  |  |
|  | Any antenatal care | | Complete antenatal care | | Postnatal care | | Facility-based delivery | | Vaccinations | | Sick child visit | |
|  | RR | p | RR | p | RR | p | RR | p | RR | p | RR | p |
| PHCPI quality | 1.99 | 0.003** | 2.33 | 0.007** | 3.11 | 0.034* | 0.73 | 0.63 | 5.44 | 0.002** | 1.78 | 0.42 |
| Poverty | 0.96 | 0.006** | 0.82 | 0.000*** | 0.94 | 0.27 | 0.55 | 0.000*** | 0.89 | 0.026* | 0.79 | 0.002** |
| Married or cohabitating | 1.01 | 0.49 | 1.05 | 0.19 | 1.12 | 0.07 | 1.02 | 0.83 | 0.97 | 0.54 | 1.08 | 0.41 |
| Age | 1.02 | 0.044* | 1.04 | 0.003** | 1.06 | 0.005** | 0.95 | 0.06 | 1.07 | 0.002** | 0.98 | 0.46 |
| Age Squared | 1.00 | 0.042* | 1.00 | 0.008** | 1.00 | 0.006** | 1.00 | 0.12 | 1.00 | 0.008** | 1.00 | 0.61 |
| Women's education |  |  |  |  |  |  |  |  |  |  |  |  |
| No education | Ref |  | Ref |  | Ref |  | Ref |  | Ref |  | Ref |  |
| Primary | 1.11 | 0.000*** | 1.22 | 0.000*** | 1.13 | 0.032* | 1.69 | 0.000*** | 1.17 | 0.003** | 1.19 | 0.07 |
| Secondary | 1.17 | 0.000*** | 1.45 | 0.000*** | 1.32 | 0.000*** | 3.00 | 0.000*** | 1.29 | 0.000*** | 1.52 | 0.000*** |
| Higher | 1.17 | 0.001** | 1.59 | 0.000*** | 1.53 | 0.027* | 4.74 | 0.000*** | 1.45 | 0.044* | 1.57 | 0.29 |
| Constant | 0.42 | 0.000*** | 0.16 | 0.000*** | 0.07 | 0.000*** | 0.54 | 0.306 | 0.05 | 0.000*** | 0.32 | 0.06 |
| N | 3230 |  | 3230 |  | 3230 |  | 3236 |  | 3601 |  | 2545 |  |
|  |  |  |  |  |  |  |  |  |  |  |  |  |
| PHCPI quality and utilization in urban households | | | | |  |  |  |  |  |  |  |  |
|  | Any antenatal care | | Complete antenatal care | | Postnatal care | | Facility-based delivery | | Vaccinations | | Sick child visit | |
|  | RR | p | RR | p | RR | p | RR | p | RR | p | RR | p |
| PHCPI quality | 1.14 | 0.34 | 0.98 | 0.94 | 0.72 | 0.64 | 0.87 | 0.80 | 2.56 | 0.07 | 1.12 | 0.85 |
| Poverty | 0.87 | 0.08 | 0.84 | 0.13 | 0.93 | 0.65 | 0.72 | 0.06 | 0.68 | 0.09 | 0.89 | 0.026* |
| Married or cohabitating | 1.02 | 0.46 | 1.04 | 0.29 | 1.09 | 0.23 | 0.94 | 0.25 | 1.09 | 0.27 | 0.97 | 0.54 |
| Age | 0.99 | 0.29 | 1.03 | 0.15 | 0.99 | 0.72 | 0.97 | 0.32 | 1.13 | 0.001*** | 1.07 | 0.002** |
| Age Squared | 1.00 | 0.27 | 1.00 | 0.29 | 1.00 | 0.60 | 1.00 | 0.26 | 1.00 | 0.004** | 1.00 | 0.008** |
| Women's education |  |  |  |  |  |  |  |  |  |  |  |  |
| No education | Ref |  | Ref |  | Ref |  | Ref |  | Ref |  | Ref |  |
| Primary | 1.17 | 0.004** | 1.31 | 0.000*** | 1.06 | 0.59 | 1.13 | 0.31 | 1.27 | 0.07 | 1.47 | 0.032* |
| Secondary | 1.27 | 0.000*** | 1.62 | 0.000*** | 1.09 | 0.43 | 1.77 | 0.000*** | 1.46 | 0.004** | 1.66 | 0.004** |
| Higher | 1.31 | 0.000*** | 1.80 | 0.000*** | 1.25 | 0.08 | 2.46 | 0.000*** | 1.63 | 0.000*** | 2.03 | 0.001** |
| Constant | 0.79 | 0.16 | 0.31 | 0.000*** | 0.52 | 0.27 | 0.62 | 0.37 | 0.02 | 0.000*** | 0.10 | 0.008** |
| N | 1618 |  | 1618 |  | 1618 |  | 1461 |  | 1636 |  | 1126 |  |

Appendix 4: Sensitivity results

| **Larger buffer** | |  | |  | | |  | | | |  | | |  | | |  | | |  |  |
| --- | --- | --- | --- | --- | --- | --- | --- | --- | --- | --- | --- | --- | --- | --- | --- | --- | --- | --- | --- | --- | --- |
| Table 1. Association between urban service utilization and quality with larger buffer, adjusting for poverty, education, married, age, age squared | | | | | | | | | | | | | | | | | | | | |  |
|  | Infrastructure quality | | | | | | | | | Service delivery quality | | | | | | | | |  |  |  |
|  | RR | | p | | | N | | | RR | | | | p | | | N | |  |  |  |  |
| Any ANC | 0.87 | | 0.243 | | | 1618 | | | 1.35 | | | | 0.082 | | | 1618 | |  |  |  |  |
| Complete ANC | 0.97 | | 0.859 | | | 1618 | | | 1.47 | | | | 0.209 | | | 1618 | |  |  |  |  |
| Facility delivery | 2.15 | | 0.024* | | | 1461 | | | 0.72 | | | | 0.632 | | | 1461 | |  |  |  |  |
| PNC | 3.46 | | 0.005** | | | 1618 | | | 1.26 | | | | 0.767 | | | 1618 | |  |  |  |  |
| Complete vaccinations | 2.37 | | 0.038* | | | 1636 | | | 5.80 | | | | 0.000*** | | | 1636 | |  |  |  |  |
| Sick child visit | 1.03 | | 0.947 | | | 1126 | | | 0.91 | | | | 0.881 | | | 1126 | |  |  |  |  |
|  |  | |  | | |  | | |  | | | |  | | |  | |  |  |  |  |
| Table 2. Association between rural service utilization and quality with larger buffer, adjusting for poverty, education, married, age, age squared | | | | | | | | | | | | | | | | | | | | |  |
|  | | Infrastructure quality | | | | | | | | | Service delivery quality | | | | | | | | |  |  |
|  | | RR | | p | | | N | | | | RR | | | p | | | N | | |  |  |
| Any ANC | | 1.37 | | 0.034* | | | 3230 | | | | 2.05 | | | 0.020* | | | 3230 | | |  |  |
| Complete ANC | | 2.07 | | 0.012* | | | 3230 | | | | 3.68 | | | 0.032* | | | 3230 | | |  |  |
| Facility delivery | | 4.22 | | 0.000*** | | | 3236 | | | | 0.68 | | | 0.671 | | | 3236 | | |  |  |
| PNC | | 2.76 | | 0.014* | | | 3230 | | | | 2.65 | | | 0.283 | | | 3230 | | |  |  |
| Complete vaccinations | | 2.85 | | 0.002** | | | 3601 | | | | 5.76 | | | 0.064 | | | 3601 | | |  |  |
| Sick child visit | | 0.95 | | 0.917 | | | 2545 | | | | 5.53 | | | 0.13 | | | 2545 | | |  |  |
|  | |  | |  | | |  | | | |  | | |  | | |  | | |  |  |
| **Best facility in buffer** | |  | |  | | |  | | | |  | | |  | | |  | | |  |  |
| Table 3. Association between urban service utilization and best quality in buffer, adjusting for poverty, education, married, age, age squared | | | | | | | | | | | | | | | | | | | | |  |
|  | | Best infrastructure quality | | | | | | | | | Best service delivery quality | | | | | | | | |  |  |
|  | | RR | | p | | | N | | | | RR | | | p | | | N | | |  |  |
| Any ANC | | 0.82 | | 0.003** | | | 1618 | | | | 0.74 | | | 0.013* | | | 1618 | | |  |  |
| Complete ANC | | 0.82 | | 0.14 | | | 1618 | | | | 0.81 | | | 0.42 | | | 1618 | | |  |  |
| Facility delivery | | 1.50 | | 0.12 | | | 1461 | | | | 1.44 | | | 0.47 | | | 1461 | | |  |  |
| PNC | | 1.92 | | 0.042* | | | 1618 | | | | 0.41 | | | 0.11 | | | 1618 | | |  |  |
| Complete vaccinations | | 1.01 | | 0.98 | | | 1636 | | | | 0.72 | | | 0.55 | | | 1636 | | |  |  |
| Sick child visit | | 1.36 | | 0.30 | | | 1126 | | | | 1.47 | | | 0.54 | | | 1126 | | |  |  |
|  | |  | |  | | |  | | | |  | | |  | | |  | | |  |  |
| Table 4. Association between urban service utilization and best quality in buffer, adjusting for poverty, education, married, age, age squared | | | | | | | | | | | | | | | | | | | | |  |
|  | | Best infrastructure quality | | | | | | | | | Best service delivery quality | | | | | | | | |  |  |
|  | | RR | | p | | | N | | | | RR | | | p | | | N | | |  |  |
| Any ANC | | 1.20 | | 0.016* | | | 3230 | | | | 1.69 | | | 0.008** | | | 3230 | | |  |  |
| Complete ANC | | 1.20 | | 0.17 | | | 3230 | | | | 2.02 | | | 0.034* | | | 3230 | | |  |  |
| Facility delivery | | 1.83 | | 0.011* | | | 3236 | | | | 1.30 | | | 0.70 | | | 3236 | | |  |  |
| PNC | | 2.18 | | 0.000*** | | | 3230 | | | | 3.43 | | | 0.007** | | | 3230 | | |  |  |
| Complete vaccinations | | 1.14 | | 0.46 | | | 3601 | | | | 0.72 | | | 0.50 | | | 3601 | | |  |  |
| Sick child visit | | 0.94 | | 0.78 | | | 2545 | | | | 1.94 | | | 0.24 | | | 2545 | | |  |  |
|  | |  | |  | | |  | | | |  | | |  | | |  | | |  |  |
| **Pregnancies in the past two years** | | | | |  | | |  | | | |  | | |  | | |  | | | |
| Table 5. Association between 2-year service utilization and quality in urban areas, adjusting for poverty, education, married, age, age squared | | | | | | | | | | | | | | | | | | | | |  |
|  | | Infrastructure quality | | | | | | | | | Service delivery quality | | | | | | | | |  |  |
|  | | RR | | p | | | N | | | | RR | | | p | | | N | | |  |  |
| Any ANC | | 1.05 | | 0.79 | | | 477 | | | | 2.01 | | | 0.011* | | | 477 | | |  |  |
| Complete ANC | | 0.65 | | 0.22 | | | 477 | | | | 1.60 | | | 0.39 | | | 477 | | |  |  |
| Facility delivery | | 1.56 | | 0.26 | | | 424 | | | | 0.93 | | | 0.92 | | | 424 | | |  |  |
| PNC | | 1.24 | | 1.15 | | | 477 | | | | 1.47 | | | -0.57 | | | 477 | | |  |  |
|  | |  | |  | | |  | | | |  | | |  | | |  | | |  |  |
| Table 6. Association between 2-year service utilization and quality in rural areas, adjusting for poverty, education, married, age, age squared | | | | | | | | | | | | | | | | | | | | |  |
|  | | Infrastructure quality | | | | | | | | | Service delivery quality | | | | | | | | |  |  |
|  | | RR | | p | | | N | | | | RR | | | p | | | N | | |  |  |
| Any ANC | | 1.37 | | 0.005** | | | 1134 | | | | 2.11 | | | 0.020* | | | 1134 | | |  |  |
| Complete ANC | | 2.36 | | 0.20 | | | 1134 | | | | 2.36 | | | 0.10 | | | 1134 | | |  |  |
| Facility delivery | | 1.22 | | 0.005** | | | 1131 | | | | 1.22 | | | 0.83 | | | 1131 | | |  |  |
| PNC | | 2.74 | | 0.13 | | | 1134 | | | | 2.74 | | | 0.23 | | | 1134 | | |  |  |
